# How Can Hearing Loss Cause Dementia?

**DOI:** 10.1016/j.neuron.2020.08.003

**Published:** 2020-11-11

**Authors:** Timothy D. Griffiths, Meher Lad, Sukhbinder Kumar, Emma Holmes, Bob McMurray, Eleanor A. Maguire, Alexander J. Billig, William Sedley

**Affiliations:** 1 Biosciences Institute, Newcastle University Medical School, Newcastle upon Tyne NE2 4HH, UK; 2 Wellcome Centre for Human Neuroimaging, UCL Queen Square Institute of Neurology, University College London, London WC1N 3AR, UK; 3 Human Brain Research Laboratory, Department of Neurosurgery, University of Iowa Hospitals and Clinics, Iowa City, IA 52242, USA; 4 Departments of Psychological and Brain Sciences, Communication Sciences and Disorders, Otolaryngology, University of Iowa, Iowa City, IA 52242, USA; 5 UCL Ear Institute, University College London, London WC1X 8EE, UK

**Keywords:** dementia, hearing loss, medial temporal lobe, Alzheimer disease, auditory cognition

## Abstract

Epidemiological studies identify midlife hearing loss as an independent risk factor for dementia, estimated to account for 9% of cases. We evaluate candidate brain bases for this relationship. These bases include a common pathology affecting the ascending auditory pathway and multimodal cortex, depletion of cognitive reserve due to an impoverished listening environment, and the occupation of cognitive resources when listening in difficult conditions. We also put forward an alternate mechanism, drawing on new insights into the role of the medial temporal lobe in auditory cognition. In particular, we consider how aberrant activity in the service of auditory pattern analysis, working memory, and object processing may interact with dementia pathology in people with hearing loss. We highlight how the effect of hearing interventions on dementia depends on the specific mechanism and suggest avenues for work at the molecular, neuronal, and systems levels to pin this down.

## Teaser

Griffiths et al. evaluate candidate explanations for the relationship between hearing loss and dementia. As well as considering common pathology, impoverished input, and resource competition, they develop a novel account based on specific interactions between auditory cognition and dementia pathology in the medial temporal lobe.

## Introduction

Hearing loss in midlife has been estimated to account for 9% of cases of dementia, a huge (but potentially reversible) disease burden given that dementia affects 47 million people worldwide (Livingston et al., 2017). Acquired hearing loss is most commonly caused by cochlear damage, while dementia is due to cortical degeneration that typically begins in multimodal cortex. This immediately begs the question of how the two are linked. This is a crucial question from a theoretical perspective, as there are multiple biological and psychological pathways that may link peripheral auditory function to broad-based cortical changes associated with dementia. It also has critical practical implications because while it is difficult, if not impossible, to remediate cortical degradation, hearing loss is widely treatable with hearing aids or cochlear implants. Thus, an understanding of the mechanisms linking the two could have wide-ranging public health importance.

The aim of this article is to examine brain bases for the relationship between hearing loss and dementia. We appraise mechanisms based on common pathology in the cochlea and brain, deterioration of brain resources due to an impoverished acoustic environment, and the diminished availability of cognitive resources that are occupied in support of listening in difficult conditions. A novel mechanism that we propose here, however, is based on a critical interaction between auditory cognitive processing in the medial temporal lobe (MTL) and dementia pathology. This suggested basis is informed by recent work on the role of the MTL in auditory cognition and our current understanding of the relationship between brain activity and the molecular underpinnings of dementia.

## Evidence

Mounting evidence supports a link between hearing loss and dementia. Loughrey et al. (2018) present a meta-analysis of 36 studies that measured cognitive function and pure tone audiometry, the majority of which were cross-sectional. This analysis demonstrated weak but significant associations between hearing loss and both cognitive impairment and dementia. Three studies (Deal et al., 2017; Gallacher et al., 2012; Lin et al., 2011) followed subjects after midlife hearing assessment to evaluate the incidence of dementia longitudinally. Livingston et al. (2017) combined them in a meta-analysis as all three used an objective measure (pure-tone audiometry), followed up subjects for >5 years, and accounted for other possible risk factors. This gave an estimate of the relative risk of incident dementia due to hearing loss of 1.94 (1.38–2.73), after accounting for other factors.

**Figure 1:**
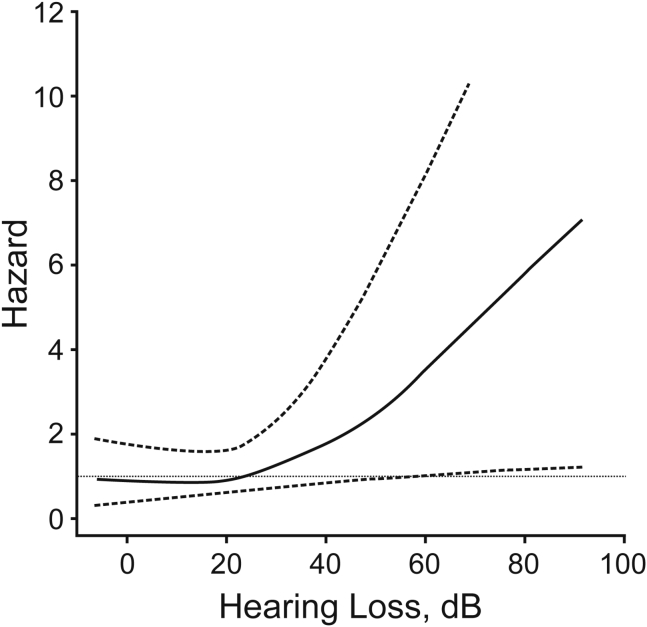
Risk of Incident Dementia (Hazard Ratio) as a Function of Hearing Loss The plotted hazard ratio accounts for other risk factors. Reproduced with permission from Lin, F.R., Metter, E.J., O’Brien, R.J., Resnick, S.M., Zonderman, A.B., and Ferrucci, L. (2011). Hearing loss and incident dementia. *Arch. Neurol*. 68, 214–220. Copyright © 2011 American Medical Association. All rights reserved.

The original study used in the longitudinal meta-analysis is instructive (Lin et al., 2011). It was based on 639 subjects with a wide age range (36–90 years of age) followed up for 10 years after pure-tone audiometry. The analysis established the risk of dementia as a function of hearing loss at the initial measurement point, after adjusting for a large number of demographic and health factors, including age, sex, race, education, diabetes mellitus, smoking, and hypertension (Figure 1). Fifty-eight cases of dementia developed, 37 (63%) of which were due to Alzheimer disease (AD). The other two studies used in the longitudinal meta-analysis also demonstrate a stratified risk as a function of hearing loss. In the other study of incident dementia that assessed dementia subtypes (Gallacher et al., 2012), 41 of 79 incident cases (51%) were nonvascular dementia (most of which met the criteria for AD; McKhann et al., 1984) and 38 incident cases were vascular.

Models of the brain mechanism by which hearing loss is linked to dementia must account for the epidemiological findings: an increase in the risk of developing dementia as a function of hearing loss in midlife, even after accounting for vascular risk factors, in which the cases generally meet the criteria for AD or vascular dementia.

## Mechanisms

**Figure 2:**
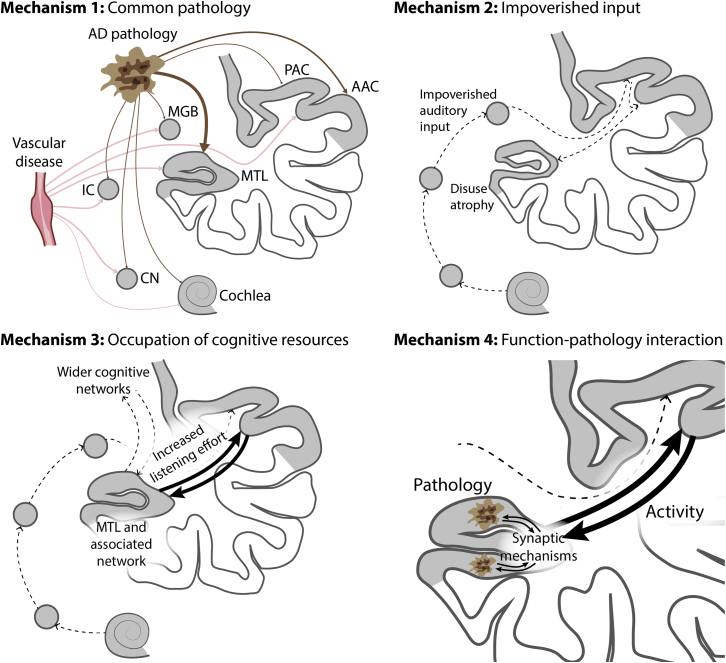
Possible Mechanisms for Dementia Related to Hearing Loss Mechanism 1: common pathology due to Alzheimer disease (AD) or vascular disease affects the cochlea and/or the ascending pathway (causing hearing loss) and MTL (causing dementia). Mechanism 2: impoverished environment caused by hearing loss leads to altered brain structure in the auditory cortex and hippocampus and decreased cognitive reserve, and therefore decreased resilience to dementia. Mechanism 3: increased brain activity in the MTL and a wider network during speech-in-noise analysis competes for the resources within that network that are also needed for other aspects of higher cognition. We argue in the text that this may be a better model for cognitive deficits in elderly people due to hearing loss as opposed to dementia per se. Mechanism 4: interaction between altered activity related to pattern analysis in the MTL during difficult listening and the pathology of AD. The model is based on the same mechanism for increased activity as mechanism 3, but it differs in the incorporation of a specific interaction with the molecular bases of AD. This is based on an interaction between increased activity and synaptic changes associated with AD. We also consider a mechanism in the text due to decreased activity interacting with AD pathology (not shown here). AAC, auditory association cortex; CN, cochlea nucleus; IC, inferior colliculus; MGB, medial geniculate body; MTL, medial temporal lobe; PAC, primary auditory cortex.

There are a number of possible mechanisms for the relationship between hearing loss and dementia, summarized in Figure 2. These are not mutually exclusive, and we consider the strength of support for each from the available evidence.

### Mechanism 1: Common Pathology

A first possible mechanism is common pathology affecting the cochlea and ascending auditory pathway (causing hearing loss) and the cortex (causing dementia). AD-related pathology has been observed in the retina (Hadoux et al., 2019), but it is not well established as occurring in the cochlea. Transgenic mouse models of AD suggest that AD may be associated with cochlear pathology and hearing loss, but the loss is early onset (Liu et al., 2020; O’Leary et al., 2017; Omata et al., 2016; Wang and Wu, 2015), unlike the midlife impairment in humans mentioned above. In humans, pathological changes related to AD have been described in nuclei in the ascending auditory pathway (Baloyannis et al., 2009; Parvizi et al., 2001; Sinha et al., 1993). Pathological changes also occur in the auditory cortex (Baloyannis et al., 2011; Lewis et al., 1987), with limited data suggesting a relative sparing of primary auditory cortex by the disease process as compared to higher auditory areas (Esiri et al., 1986). However, hearing loss due to brainstem or cortical pathology is uncommon and is generally associated with obvious macroscopic lesions (Griffiths et al., 2010), as opposed to the more subtle microscopic changes described in these studies. Moreover, the hearing loss demonstrated in the studies relating it to dementia is typically pronounced at high frequencies, which is consistent with age-related deterioration in the cochlea rather than damage to the central pathways caused by AD.

Vascular pathology can also occur in the cochlea, and this is one of the factors implicated in typical acquired hearing loss (Kurata et al., 2016). It can also affect the ascending auditory pathway and auditory cortex. Vascular mechanisms are therefore potential contributors to the hearing loss associated with cases of vascular dementia. However, the meta-analysis of incident dementia cases demonstrates a relationship that survives correction for vascular risk factors (Livingston et al., 2017), and the majority of incident cases are due to AD.

### Mechanism 2: Impoverished Environment Causing Decreased Cognitive Reserve

A second possible mechanism is that hearing loss leads to the decreased stimulation of cognitive processing. The idea is that auditory deprivation creates an impoverished environment, particularly with the diminishment of speech and language input, that negatively affects brain structure and function. This change in brain structure and function is a risk factor for the subsequent development of dementia.

A large number of animal studies, mainly on rodents, have demonstrated changes in brain and behavior deriving from the experience of enriched (as opposed to impoverished) environments. Structural changes can be seen macroscopically (cortical thickness) and at the level of synapses, dendrites, somata, axons, glia, and vasculature (reviewed in Markham and Greenough (2004)). Based on animal models, it has been proposed (Nithianantharajah and Hannan, 2009; Sale et al., 2014) that such changes may establish cognitive reserve, which is argued to protect against dementia in humans (Stern, 2012).

In terms of human behavior, the idea of an impoverished acoustic environment immediately raises the question of which aspects of the environment are impoverished in subjects with hearing loss. People with impaired hearing receive less stimulation from the acoustic environment: critically, a distorted peripheral representation of sounds means that they also they have less access to verbal and emotional information in speech, a critical mediator of complex social interactions for most people. One possibility is that this lack of verbal and emotional stimulation negatively affects brain structure and function directly. Alternatively, an effect of impaired speech perception on dementia may be mediated by a reduction in the quality of social interactions in which the individual engages. Poor social interactions are a risk factor for dementia that emerges in later life, with a similar risk as smoking and inactivity (Kuiper et al., 2015; Livingston et al., 2017). Access to speech is particularly problematic in noisy environments, which are commonplace in everyday social settings and especially challenging for people with hearing loss (Gatehouse and Noble, 2004). These difficulties in perceiving speech can lead to social withdrawal (Hughes et al., 2018b), which may exert a direct or compounding negative effect on brain structure and function.

However, our understanding of the links between these issues and dementia is limited by the fact that most human studies on this subject rely on pure-tone audiometry to assess auditory function. This standard clinical tool of the audiologist identifies the minimum sound level at which a listener can detect tones of various frequencies. It has demonstrated a relationship between these levels and dementia, such as that shown in Figure 1. The relationship between pure-tone audiometry and speech-in-noise measures is complex (see Holmes and Griffiths, 2019 for discussion), as speech-in-noise processing requires more than simply detecting quiet sounds. A number of studies directly assessing speech-in-noise ability have suggested a relationship with cognitive performance or with the development of dementia (Häggström et al., 2020; Jalaei et al., 2019; Mamo et al., 2019; Nixon et al., 2019; Pronk et al., 2019). However, the ability to make clear causal claims is problematic because speech-in-noise ability requires a host of cognitive processes—auditory attention and grouping, word recognition, sentence processing, and sometimes speech production—that may themselves be affected by dementia. Further large studies are required to establish whether incident dementia is better determined by hearing loss or speech-in-noise ability, the degree to which cognitive components of speech-in-noise may individually be affected by dementia, and whether such effects are modulated by social factors.

A variety of lines of evidence suggest that listening experience may have a direct impact on the human brain. In parallel to the enriched environment studies with mice, the active listening experience of musicians is associated with positive effects on the structure of auditory cortex and the hippocampus (Hyde et al., 2009; Luders et al., 2004; Schlaug et al., 1995) and functional changes in the hippocampus (Herdener et al., 2010). Piano tuners, expert listeners who spend large amounts of time carrying out a highly specialized form of selective listening, demonstrate hippocampal structural correlates of that experience (Teki et al., 2012).

More directly relevant to the issue of hearing loss, several studies have demonstrated a relationship between the macroscopic structure of auditory cortex and acquired hearing loss (Eckert et al., 2012; Neuschwander et al., 2019; Peelle et al., 2011; Profant et al., 2014). However, this seems an unlikely basis for a decrease in cognitive reserve that otherwise protects against dementia; dementia typically manifests first as an impairment of high-level cognitive function, with pathology in multimodal cortex. In contrast, a recent human study (Armstrong et al., 2019) highlighted increases in volume decline with hearing loss in MTL areas that are more prominent targets for AD pathology (Braak and Braak, 1991). The study demonstrated greater volume decline in the hippocampus and entorhinal cortex as a function of midlife hearing loss (45–65 years of age) measured ∼20 years before the scans. This suggests a possible neural mediator of the link between hearing loss and dementia.

### Mechanism 3: Increased Cognitive Resources Needed for Listening

A third mechanism is based on the idea that people with hearing impairment use greater cognitive resources for listening, making these resources unavailable for other aspects of higher cognition when they are “occupied” during listening. “Resources” refers here to the means for cognitive tasks such as attention (Kahneman, 1973), working memory (Manohar et al., 2019), or language processing (Pichora-Fuller et al., 2016). There is debate about how cognitive resources are allocated, and the corresponding neural bases. With respect to working memory, for example, there is a question about the extent to which resources may be specifically allocated to objects or represent a distributed resource (Bays and Husain, 2008; Kumar et al., 2013). Further debate concerns the extent to which working memory resources reflect neuronal or synaptic mechanisms, or both (Manohar et al., 2019). What is important here, however, is that there is a fixed capacity for many general cognitive operations. These resources may be absorbed when listening becomes challenging, reducing their availability for other aspects of cognition.

Mechanisms 2 and 3 may at first appear at odds: in mechanism 2, the problem is the decreased stimulation of auditory cognitive networks, while in mechanism 3, there is increased stimulation. The critical difference is that mechanism 2 leads to changes in neuronal mechanisms and brain structure before the onset of dementia, causing an increased risk of subsequent dementia, while mechanism 3 is based on changes in brain activity during dementia that may explain the cognitive deficits.

Behaviorally, there is good evidence that listening difficulty decreases the cognitive resources that are available for other activities. This is widely seen in dual-task paradigms (29 studies are reviewed in Gagné et al., 2017). Here, it is commonly observed that while listening to speech (particularly in noise), participants demonstrate a decrease in performance in a secondary non-auditory cognitive task. Critically, the degree of dual-task interference increases when speech is masked or degraded, potentially simulating the challenges of hearing-impaired listeners. The studies have used a variety of secondary tasks, including tactile recognition, visuomotor tracking, speeded visual and verbal target detection, visual working memory, and the Stroop task (Gagné et al., 2017). However, the secondary tests do not include delayed memory, the key deficit in typical AD, which would be difficult to incorporate into these paradigms. This makes it hard to draw a direct link to dementia. In their support, however, the commonly used tests are attentionally demanding, as is the case for delayed memory. This suggests an attentional basis by which the demands of speech-in-noise listening may affect memory function in typical early AD.

Listening to speech under difficult conditions, when subjects have hearing impairment, therefore has a likely acute effect on cognitive domains that are deficient in dementia. In terms of the brain basis for speech perception under difficult listening conditions, meta-analyses of functional imaging studies (Adank, 2012; Alain et al., 2018) demonstrate the involvement of auditory cortex and the left inferior frontal lobe. Individual studies have identified the involvement of other neocortical areas (Binder et al., 2004; Davis et al., 2011; Du et al., 2016; Eckert et al., 2016; Hill and Miller, 2010; Scott et al., 2004; Vaden et al., 2016; Zekveld et al., 2006) and the hippocampus (Bishop and Miller, 2009; Blank et al., 2018; Davis et al., 2011).

The behavioral data therefore support the greater use of cognitive resources during listening in difficult conditions, and imaging studies support the use of a broad network of areas, including auditory cortex, the language network, and the hippocampus. The possible relevance of these observations to the development of dementia cannot be dismissed. However, these resources appear to be used in the short term, especially when listening to difficult speech, not when speech is easy (or when the subject is not actively listening or no longer “trying” to listen). The question thus arises as to how the increased use of these resources in the short term contributes to the development of dementia, defined as a cognitive phenotype with specific and enduring pathology, generally based on testing with single tasks. In other words, how does the interfering effect of speech in noise on cognition persist and affect cognitive tests for dementia that are generally carried out without any distracting task? In our view, this mechanism is a more compelling basis for hearing loss as a potential cause for the wide range of cognitive deficits in elderly people (considered in Wayne and Johnsrude, 2015) that can occur in the absence of the specific molecular and neuronal pathology of AD. In the case of cognitive deficits in the elderly, the idea is that elderly people with hearing impairment are forced continually to carry out a dual task, which impairs cognition. Even then, there is a question as to how such immediate effects translate to a persistent impact on cognition when this is tested with single tasks, particularly non-auditory ones (Wayne and Johnsrude, 2015).

### Mechanism 4: Interaction between Brain Activity Related to Auditory Cognition and Dementia Pathology

The previous mechanism considered the widespread brain resources used for speech-in-noise listening. A fourth possible explanation focuses on auditory cognitive mechanisms in the MTL that may be specifically linked to AD pathology in the same region. This mechanism starts from the same idea as mechanism 3, that hearing loss alters cortical activity, including in the MTL. The critical difference from mechanism 3 is the incorporation of an interaction between that altered activity and AD pathology.

The AD pathology that best correlates with the cognitive phenotype is neurofibrillary change related to tau pathology (Jack et al., 2019; Morris and Price, 2001; Pereira et al., 2019). The earliest neurofibrillary changes in typical AD are found in MTL structures, particularly the perirhinal cortex, which has a strong functional relationship to the hippocampus (Braak and Braak, 1991; Khan et al., 2014). This raises the possibility of an interaction between this pathological process and changes in neuronal activity in MTL structures that occur in hearing impaired individuals.

Although MTL structures are not classically regarded as part of the auditory system, animal models support their role in auditory processing. A number of studies have used trace conditioning with rabbits and rodents, requiring memory for sounds for up to tens of seconds, and implicating the hippocampus as critical for this learning (Ahmed et al., 2020; Solomon et al., 1986). Disconnecting input to the rodent hippocampus also disrupts memory for the duration and presentation rate of click trains (Meck et al., 1984). In another rodent study, single-unit recordings from the hippocampus identified cells that were selectively tuned within a simple acoustic feature space (Aronov et al., 2017). Critically, that tuning was only present when the animals carried out an active task requiring responses to the feature space. Moreover, this report also found hippocampal units that responded to single points in both physical space and acoustic frequency (by definition, also “place cells”) and entorhinal units that responded to multiple points in both physical space and acoustic frequency (by definition, also “grid cells”).

The human hippocampus is active during a number of types of sound analysis. It is implicated in a generative model for the recognition of patterns that emerge from a random sequence (Barascud et al., 2016) and in the statistical learning of streams of tones (Covington et al., 2018; Schapiro et al., 2014). The human hippocampus is also involved in working memory processes that are required for the analysis of acoustic patterns that evolve over time. Functional imaging (Kumar et al., 2016) has demonstrated increased blood-oxygen-level-dependent (BOLD) activity in the hippocampus when subjects hold tones in mind, and human local field potential recordings (Kumar et al., 2020) have demonstrated low-frequency oscillatory activity during the same process. Representations of auditory content are also available to the hippocampus during active listening. This has been demonstrated by successful decoding of the identity of specific tone clouds (Kumar et al., 2014) from hippocampal multivoxel BOLD activity patterns. Hippocampal activity patterns specific to particular sequences of spoken letters have also been inferred using similar techniques (Kalm et al., 2013). These human studies suggest the use of computational mechanisms in the MTL for the active analysis of acoustic patterns.

The idea of auditory pattern analysis involving MTL mechanisms is congruent with visual work suggesting a role for these in perception. Non-human primate lesion work implicates the perirhinal cortex in disambiguating similar visual objects (Buckley et al., 2001; Bussey et al., 2002). Human studies support a role for the perirhinal cortex in object invariance (Erez et al., 2016). Studies of human subjects with damage to the perirhinal cortex have shown deficits in the separation of objects or visual figure-ground analysis (Barense et al., 2012) and the assessment of objects within scenes (Yeung et al., 2019). Other lines of evidence, for example, from Mullally et al. (2012), implicate the hippocampus in the “construction” of visual scenes during both perception and memory. Finally, a series of studies on hippocampally lesioned patients show performance decrements in a variety of complex visual tasks, even when there are no demands on long-term memory (Warren et al., 2011, 2012, 2014).

A model for visual analysis in the MTL has been proposed based on the conjunction of sensory and conceptual features of objects in the perirhinal cortex (Martin et al., 2018). Another model has incorporated pattern analysis by the hippocampus into the mechanism for episodic memory (Liu et al., 2016). The idea is that the hippocampus separates patterns of inputs during encoding and completes patterns during retrieval to allow recall, even of partial or degraded representations. A number of studies have addressed this idea, reviewed by Liu et al. (2016), and the dentate gyrus and area CA3 have been suggested as possible computational bases (Rolls, 2013; Sakon and Suzuki, 2019). In vision, there is therefore a range of evidence for the involvement of the perirhinal cortex and hippocampus in the analysis of objects within the scene. However, the concepts of object and scene can also be applied to auditory analysis (Bizley and Cohen, 2013; Bregman, 1990; Griffiths and Warren, 2004) and the analysis of speech in noise can be regarded as a form of figure-ground analysis. Critically, the discovery of auditory “place” and “grid cells” that also carry out auditory analysis (Aronov et al., 2017) strengthens the idea of common computational resources for the active analysis of the visual and acoustic world.

What changes can be driven by hearing loss in MTL neural mechanisms, which, as we described, are vulnerable to early AD pathology? One possibility is the altered activity of auditory cognitive mechanisms for the analysis of acoustic patterns during speech-in-noise perception when listening is challenging. Studies support the involvement of the hippocampus in the analysis of degraded speech (Bishop and Miller, 2009; Blank et al., 2018; Davis et al., 2011). Initial data support a role for the kinds of complex acoustic pattern analysis mechanisms described above in speech-in-noise perception. A critical function for speech-in-noise analysis is auditory figure-ground processing, the ability to extract a target auditory object from background noise. This mechanism can be isolated using non-linguistic stimuli (a persistent complex figure on a background of random tones). Analysis of such stimuli correlates with speech-in-noise ability (Holmes and Griffiths, 2019). The question is whether this cognitive mechanism engages the hippocampus. Initial imaging studies using similar stimuli have not demonstrated its involvement (Teki et al., 2011, 2016). However, these did not require an active task, which the visual literature suggests may be necessary; further work is needed. However, supporting a role of the hippocampus in pattern analysis, auditory working memory for tone frequency engages the hippocampus (Kumar et al., 2016, 2020) and correlates with speech-in-noise ability (Lad et al., 2020). Phonological working memory also correlates with speech-in-noise ability (Akeroyd, 2008), although there has been debate about this (Füllgrabe and Rosen, 2016). Intuitively, the correlation between working memory and speech-in-noise ability makes sense; the idea is that during speech-in-noise listening, auditory objects with similar features can be linked by working memory to facilitate the separation of foreground objects of interest.

Auditory-pattern analysis and working-memory mechanisms may become more active when hearing loss increases the difficulty of segregation of speech from noise. We consider above the fundamental mechanisms for auditory cognition that explain increased MTL activity in studies of degraded speech (Bishop and Miller, 2009; Blank et al., 2018; Davis et al., 2011). The increased activity in the MTL and wider cognitive network are easily incorporated into predictive coding models (see Sedley et al., 2016 for a discussion of auditory models) in which imprecise signals from auditory cortex to the higher centers are associated with greater activity in the higher centers that generate perceptual predictions and increased backward predictions to auditory cortex. A number of lines of evidence suggest interactions between AD pathology and neuronal activity at the level of synapses, neurons, or networks (Bero et al., 2011; de Haan et al., 2012; Ittner et al., 2010; Kocagoncu et al., 2020). We elaborate these further below.

### Interaction between Neuronal Activity and AD Pathology in the MTL

Our favored mechanism 4 is supported by circumstantial evidence—the co-occurrence of altered neuronal activity due to hearing loss and AD pathology in the MTL. We speculate here about specific links between neuronal mechanisms and AD pathology that can be tested in animal models.

A first type of interaction is based on increased neuronal activity causing or increasing AD pathology. In this case, auditory cognitive processing increases in response to a degraded input, leading to elevated neuronal activity in the MTL. A number of human studies have demonstrated the co-localization of increased activity in network hubs and tau deposition in AD (de Haan et al., 2012; Kocagoncu et al., 2020), and animal studies suggest a direct link between markers of increased neuronal activity and AD pathology (Bero et al., 2011). Here, then, degraded input leads to overactivity that causes or exacerbates AD pathology in specific regions (de Haan et al., 2012). Another possibility is that AD pathology causes increased neuronal activity in the MTL and other hubs. In rodents, tau and amyloid β affect the *N*-methyl-d-aspartic acid (NMDA) receptor (Ittner et al., 2010). This suggests a model in which the molecular pathology of AD causes changes in the function of the NMDA receptor. In turn, altered function of the NMDA receptor is a basis for excitotoxicity (Wang and Reddy, 2017), which in this case is compounded by activity during auditory cognition. Recent work on a C57BL/6 mouse model of adult hearing loss (Beckmann et al., 2020) demonstrated increases in the expression of glutamate subunits of the NMDA receptor in the hippocampus that are implicated in synaptic plasticity (long-term potentiation). Other transmitter systems implicated in synaptic plasticity were also affected. Behaviorally, and in support of the relevance of this model to AD, the mice also experienced memory loss. Overall, then, animal studies support a model based on pathologically altered synaptic mechanisms that interact with increased activity in the MTL produced by the demands of listening in difficult conditions, to cause neuronal degeneration based on excitotoxicity. Based on this, AD pathology at the molecular level alters neuronal activity, while neuronal activity causes an effect on neuronal pathology (excitotoxic cell death). If this turns out to be the case, then arguments about the causal relationship between activity and AD pathology in the MTL become specious, as this is bidirectional.

Alternatively, it could be that degraded auditory input due to hearing loss leads to reduced activity associated with auditory cognitive processes in the MTL. This is consistent with work suggesting interactions between AD pathology and activity at the level of oscillations in local networks within the MTL. Physiologically, the hippocampus exhibits different rhythms, including low-frequency theta oscillations, high-frequency gamma oscillations, and sharp wave ripples, with different hypothesized roles in mnemonic function (Colgin, 2016). Pathologically, a particular relevance of oscillations at the low end of the gamma range (25–50 Hz) related to GABAergic interneurons has been proposed in AD (Mably and Colgin, 2018). Studies in a number of mouse models of AD, reviewed by Mably and Colgin (2018), show decreases in such activity in the hippocampus. Recent work on the 5XFAD mouse AD model demonstrated that 40-Hz trains of tones drive gamma activity in auditory cortex and the hippocampus and lead to improved behavior and decreased amyloid deposition in the hippocampus (Martorell et al., 2019). Gamma activity in the MTL and neocortex is nested upon theta oscillations (Canolty et al., 2006), and any gamma-mediated effects on AD pathology may therefore be driven by alterations in theta oscillations. In turn, theta oscillations in auditory cortex show phase locking to the envelope of auditory stimuli, particularly under focused attention, but this phase locking is degraded in the presence of hearing loss, as is higher-frequency phase locking to temporal fine structure (Henry and Heinz, 2013). It is therefore worth considering whether MTL gamma rhythms may be disturbed, indirectly, through impaired stimulus phase locking in auditory cortex. Recent work suggests that entrainment to the speech envelope with electrical brain stimulation at the theta rate can improve the perception of speech-in-noise, as a potential mechanism to remediate or counteract disturbed auditory cortex phase locking (Wilsch et al., 2018).

We have speculated on two candidate causal links between auditory-related neuronal activity and AD pathology in the MTL—one based on the magnitude of activity in individual neurons associated with difficult listening and the other based on altered oscillatory activity in MTL networks due to changes in driving inputs. Both hypotheses can be tested in further animal work.

### General Comments on Mechanisms

None of the four mechanisms above can be ruled out. In our view, common pathology (mechanism 1) is a weak candidate, especially in the majority of incident dementia cases that are due to AD. The idea of a predisposition to dementia due to impoverished environment (mechanism 2) is supported by animal data that suggest precise neuronal and anatomical bases and human data that demonstrate structural changes due to hearing loss in cortex that is vulnerable to early AD. Mechanisms 3 and 4 can both be based on the increased use of general cognitive resources, with a substrate including the MTL, under difficult listening conditions. Mechanism 3 is based on increased activity alone, and is also a weak candidate basis for long-term dementia pathology. We favor mechanism 4, in which increased activity interacts with pathology in the MTL. The argument for this is based on auditory cognitive mechanisms that are relevant to difficult listening in the MTL and studies of the distribution of AD pathology. However, there is no direct evidence for the specific interaction between the two in the MTL that we propose. Finally, we should point out that the possible mechanisms are not exclusive. For example, impoverished auditory experience could cause structural changes in the MTL and a decreased cognitive reserve to protect against dementia (mechanism 2), after which there is a specific interaction between activity changes in the MTL and AD pathology (mechanism 4).

The mechanisms differ with respect to the predicted effects of intervention that can be tested in human studies (Table 1). In the case of the more plausible vascular version of mechanism 1, restoring hearing would not affect the development of vascular dementia or lead to any improvement in cognition. Mechanism 2 is based on the impoverished environment’s causing an increased risk of dementia, which arises from functional and anatomical brain changes that would not be reversed by hearing restoration. Hearing restoration could remove the increased risk if carried out early enough before such changes occur and with sufficient audiological fidelity to enable patients to engage in social interactions with a minimal amount of effort (a key barrier to engagement that many hearing-impaired patients report). Otherwise, there would still be a fixed increase in risk after restoration, dependent on the duration of hearing loss and degree of changes. Mechanism 3 is caused by an overload of cognitive resources, which could be reversed by hearing restoration, so that both risk reduction and cognitive improvement may be achieved. We argue above, however, that mechanism 3 is not a strong candidate, making this scenario unlikely. Finally, early hearing restoration could reduce the risk associated with mechanism 4 by restoring normal activity to the hippocampus. That risk would depend on the duration of hearing loss (before remediation). If the delay between initial loss and remediation were too long, then a chain of events of the sort we elaborate above may already have been set in motion to cause ongoing cortical degeneration after restoration.

**Table 1:** Effects of Hearing Intervention Predicted by Different Mechanisms

	Effect of Hearing Restoration on Dementia Risk	Cognitive Improvement Due to Hearing Restoration?
Mechanism 1: common pathology	Risk persists	No
Mechanism 2: impoverished input	Risk reduced	No
Mechanism 3: occupied cognitive resources	Risk removed	Possible
Mechanism 4: function-pathology interaction	Risk reduced	No

The predictions for mechanism 1 are based on the more likely version of mechanism 1 relating to common vascular pathology.

## Outstanding Issues

### Human Studies

We argue above for an effect of hearing loss that may be determined by an interaction between altered use of pattern analysis mechanisms in the MTL and dementia pathology. Those analysis mechanisms are used by different modalities, and one question that arises here is whether hearing loss per se is critical or whether the stimulation of those mechanisms by other modalities may overcome the increased risk. Of possible relevance is the effect of early hearing loss on dementia in prelingually Deaf signers who live in a signing environment— they have the auditory deprivation, but none of the concomitant social deprivation. Diagnosis is problematic in this group, but testing instruments exist (Atkinson et al., 2015), and more data on the prevalence of dementia in this group would be of great interest.

There is an immediate question related to the best auditory processing measures to predict the development of dementia. Further work is required to assess whether measures of hearing loss or more complex measures of auditory cognition are the critical indicators. More complex measures could include speech-in-noise and possibly synthetic stimuli that assess fundamental mechanisms for grouping, segregation, and auditory working memory that may be relevant to speech-in-noise (Barascud et al., 2016; Goll et al., 2012; Lad et al., 2020; Teki et al., 2013). However, this approach is affected by the issue of whether differences in these mechanisms cause dementia or whether these auditory cognitive mechanisms (along with many other cognitive domains) are simply affected by dementia. Very large studies would allow multivariate techniques such as structural equation modeling to account for noisy data, facilitating estimates of latent variables related to peripheral function and to central segregation and grouping that may determine the development of dementia.

A further question relates to types of dementia that are determined by hearing loss. The initial studies used standard criteria for AD and vascular dementia. Here, we developed a model based on the interaction between auditory cognitive mechanisms and dementia pathology that is only relevant to AD, making the distinction important. However, the studies of incident dementia to date have not addressed AD subtypes, and even the large studies included in the meta-analysis (Livingston et al., 2017) may be underpowered to do so. The model developed above, related to the interaction between brain activity due to auditory cognition and AD pathology, focused on the typical progression from amnesic minimal cognitive impairment affecting the MTL to established AD (McKhann et al., 1984, 2011). It is also possible, however, that auditory cognition related to brain activity in the perisylvian language regions due to the speech-in-noise effort may interact with AD pathology to cause logopenic aphasia, a form of progressive aphasia (Gorno-Tempini et al., 2008). This was not assessed in the initial studies. Finally, hearing loss may also affect the mechanisms for auditory grouping and segregation in the posterior neocortex and predispose to another AD variant: posterior cortical atrophy (Firth et al., 2019).

Increasingly, clinical work has used biomarkers of AD pathology in cerebrospinal fluid (Jack and Holtzman, 2013) and brain imaging of biomarkers (Jack et al., 2019; Morris et al., 2016; Pereira et al., 2019) to provide estimates of the extent and distribution of disease. In research studies of incident dementia related to hearing loss, amyloid and tau imaging in particular could allow the assessment of regional AD pathology related to possible models for the interaction between hearing loss and dementia pathology.

### Animal Models

Future human work has the potential to define the exact nature of the relationship between hearing loss and dementia at the level of the key predictors related to hearing loss and the resultant syndrome. Definition of the underlying neuronal and molecular mechanisms, however, requires animal models. Rodent models of AD (Götz et al., 2018) are established and allow assessment of the manipulation of the sound environment on the disease process at the behavioral and the pathological level, as in the study by Martorell et al. (2019). Rodent models of congenital deafness are well established, but more relevant models of adult deafness are increasingly becoming the focus of research (Ingham et al., 2019; Johnson et al., 2017). These are already allowing assessment of the effects of adult hearing loss on synaptic mechanisms in the MTL that may be related to AD, as above (Beckmann et al., 2020). Further work may shed light on the relationship between adult hearing loss and altered activity in the MTL and any interaction of this with AD pathology.

Rodent models of deafness and AD, however, are some distance from the patients with hearing loss who may develop dementia and are seen in the clinic. Although hippocampal and MTL organization could be relatively similar between rodents and non-human primates (NHPs) (Clark and Squire, 2013; Witter and Amaral, 2020), NHPs provide a model that is closer to humans for normal human auditory cognition (Schneider et al., 2018) and auditory cortical organization (Baumann et al., 2013). The critical connections between auditory cortex and the MTL memory system that need to be incorporated into models of hearing loss and dementia are well defined in NHPs (Munoz-Lopez et al., 2010). NHP models for AD pathology and behavior exist (Latimer et al., 2019), but only model early AD and do not allow the efficient assessment of potential causal factors provided by rodent studies of enhanced disease states. Those models may also be possible in the future; we already have a transgenic NHP model for Huntingdon disease (Yang et al., 2008), but we are some distance from a primate model that could realistically inform understanding of the relationship between hearing loss and dementia at the level of molecular, neuronal, and systems-level mechanisms.

### Treatment

We started this discussion with a claim about potentially reversible dementia due to hearing loss. This immediately raises the issue of the effect of hearing interventions on the development of dementia. Some hearing aid and cochlear implant studies have measured cognitive outcomes (Claes et al., 2018; Jayakody et al., 2017; Kalluri and Humes, 2012; Taljaard et al., 2016; Völter et al., 2018), but the follow up is generally short compared to the studies of incident dementia mentioned above, and the results are mixed. Prospective randomized controlled trials are problematic from a practical perspective, because of the low level of hearing aid use and compliance (Chien and Lin, 2012; McCormack and Fortnum, 2013), especially in middle-aged populations that may be targeted. There are also potential ethical issues, given the existing evidence related to the effect of untreated hearing loss. Prospective studies are planned, however, that have published protocols (Deal et al., 2018; Hughes et al., 2018a).

These studies of treatment are important if our fourth mechanism above is correct. Under this model, changes in brain activity related to difficult listening cause irreversible degenerative damage based on interaction with molecular pathology. In that case, the term “preventable” may be more accurate than the term “reversible,” used by the Lancet Commission (Livingston et al., 2017). A deeper understanding of the mechanism will clarify how realistic any prevention efforts may be. This will require extensive further work and debate involving epidemiologists, clinicians treating hearing loss, clinicians treating dementia, and basic scientists. We hope that precise definition of the possible mechanisms will lead to helpful further testing by basic scientists working at the molecular, neuronal, and systems levels.
